# Software and pipelines for registration and analyses of rodent brain image data in reference atlas space

**DOI:** 10.3389/fninf.2025.1629388

**Published:** 2025-09-24

**Authors:** Maja A. Puchades, Sharon C. Yates, Gergely Csucs, Harry Carey, Arda Balkir, Trygve B. Leergaard, Jan G. Bjaalie

**Affiliations:** Neural Systems Laboratory, Institute of Basic Medical Sciences, University of Oslo, Oslo, Norway

**Keywords:** spatial registration, data integration, reference atlas, software, brain, image analysis

## Abstract

Advancements in methodologies for efficient large-scale acquisition of high-resolution serial microscopy image data have opened new possibilities for experimental studies of cellular and subcellular features across whole brains in animal models. There is a high demand for open-source software and workflows for automated or semi-automated analysis of such data, facilitating anatomical, functional, and molecular mapping in healthy and diseased brains. These studies share a common need to consistently identify, visualize, and quantify the location of observations within anatomically defined regions, ensuring reproducible interpretation of anatomical locations, and thereby allowing meaningful comparisons of results across multiple independent studies. Addressing this need, we have developed a suite of desktop and web-applications for registration of serial brain section images to three-dimensional brain reference atlases (QuickNII, VisuAlign, WebAlign, WebWarp, and DeepSlice) and for performing data analysis in a spatial context provided by an atlas (Nutil, QCAlign, SeriesZoom, LocaliZoom, and MeshView). The software can be utilized in various combinations, creating customized analytical pipelines suited to specific research needs. The web-applications are integrated in the EBRAINS research infrastructure and coupled to the EBRAINS data platform, establishing the foundation for an online analytical workbench. We here present our software ecosystem, exemplify its use by the research community, and discuss possible directions for future developments.

## Introduction

Animal models are invaluable resources for exploring brain architecture, mapping the distribution of cell types and molecules in the brain, and understanding the functional roles of brain structures. When combined with genetic or other modifications for mimicking human disease mechanisms, they enable the testing of hypotheses related to health and disease. Widely accessible methods for sectioning the brain combined with microscopy techniques offer numerous benefits. They support spatial analysis since they preserve intrinsic brain architecture, and they are compatible with diverse labeling techniques to reveal specific cellular and molecular features. In tract tracing experiments, axonal tracers are injected into the brain and transported through fibers, resulting in labeling of connections (Lanciego and Wouterlood, 2020). Techniques like immunohistochemistry, immunofluorescence multiplexing, or in-situ hybridization reveal the distribution of specific cell types, gene expression patterns, and DNA sequences, for characterizing the brains of animal models.

Analysis of serial section images has traditionally involved labor-intensive manual approaches for defining anatomical regions, supplemented by quantification of features using various methods, including signal thresholding or stereological methods. Recent advancements have leveraged machine-learning based approaches for feature extraction as well as digital three-dimensional (3D) brain reference atlases. The use of 3D atlases supports a more standardized approach for defining anatomical regions, but requires accurate spatial registration between experimental section images and atlas volumes, which can be challenging to achieve (see, e.g., Bjerke et al., 2023; Yates et al., 2019).

Many open-source scripts and software are now available for researchers conducting studies on the brains of mice and rats, but they are usually tailored to different data modalities. Some address solely image registration to a reference brain atlas (Niedworok et al., 2016; Pallast et al., 2019; Tyson et al., 2022), while others offer complete analytical pipelines (Song et al., 2020; Furth et al., 2018; Chiaruttini et al., 2025; Lauridsen et al., 2022). Most are implemented in Python, Matlab, or as Fiji plugins, often requiring coding skills, which can be a barrier to a wider user base. Recent developments incorporate machine-learning to automate registration to atlases (Carey et al., 2023), aiming to enhance efficiency and reduce biases in the registration process. However, to date, such approaches are most effective with structurally intact serial sections cut in standard planes. Deep learning models still struggle with distorted or damaged tissue sections, which commonly occur. Machine-learning methods are also increasingly employed for image segmentation for identifying features to be quantified, automating and standardizing feature extraction processes (Tyson et al., 2021; Iqbal et al., 2019). While most software is developed by individual researchers to suit specific projects, there are increasing efforts to make software more accessible to the scientific community by providing user interfaces, flexible functionalities, and user documentation. Atlas-based software is thus increasingly employed in experimental studies, providing more efficient and standardized analyses compared to traditional methods [for review, see (Bjerke et al., 2023; Tyson and Margrie, 2022)]. Despite progress, it remains challenging to find combinations of user-friendly tools that can be tailored to the highly diverse needs of current research.

To facilitate standardized analyses using reference brain atlases, we here present our suite of interactive software and web-applications for atlas-based analysis of serial section images from rodent brains. The software has been designed to lower barriers for users, enabling image registration to atlases (QuickNII, VisuAlign, WebAlign, WebWarp, DeepSlice) (Carey et al., 2023; Puchades et al., 2019; Gurdon et al., 2024), diverse analyses in an atlas context (Nutil, QCAlign, LocaliZoom and MeshView) (Yates et al., 2019; Groeneboom et al., 2020), and inspection of sections and results in 2D and 3D reference space (SeriesZoom, LocaliZoom, and MeshView). The tools are interoperable, comply with open and FAIR standards for research software (Wilkinson et al., 2016; Barker et al., 2022), and are shared through and integrated in the EBRAINS research infrastructure^1^ with online user manuals and tutorials.

To demonstrate the capabilities of our software for inspecting, visualizing, and analyzing brain section images from mouse and rat, we present three examples of use from the literature comprising datasets obtained from studies with unique experimental designs and varied analytical needs (Gurdon et al., 2024; Ovsthus et al., 2024; Reiten et al., 2023). In each example, we highlight the advantages of using the EBRAINS solutions, while noting limitations and suggesting areas for future development. Although our software was developed for analysis of 2D section images, not 3D brain volumes, we also point to examples where our software has been used for analysis of 3D tissue clearing data captured by light sheet microscopy. To our knowledge, the here presented open-source software suite for serial section images is among the most comprehensive solutions currently available for experimental neuroscientists, providing an adaptable analysis ecosystem for a wide range of study paradigms.

## Methods

### Software overview

Our suite of software is modular, interoperable, and includes both desktop and web-applications for serial section images from rodent brain. The software can be used to (1) perform registration to a reference brain atlas, (2) to perform various types of analyses in the context of a reference brain atlas, and (3) to view and inspect sections and results in 2D and 3D reference space. While several of the desktop applications have been introduced in previous publications, they have been considerably updated in recent years, in response to user feedback and demands for new functionalities, and to make them compatible with additional atlases and alternative software. The web-applications are a new addition to the suite and are integrated in the EBRAINS research infrastructure and coupled to the EBRAINS data platform. They offer similar functionality to the desktop applications and were developed to simplify file management steps and to lower barriers for transparent sharing of data and results.

The suite of tools is summarized in Table 1, with software descriptions categorized by their main functional roles, links to the code repositories, and user manuals. Further technical details and scripts are found in Supplementary Table 1. The software can be combined into several analytical pipelines, with three unique pipelines described in the results and exemplified by their use in three published studies (Gurdon et al., 2024; Ovsthus et al., 2024; Reiten et al., 2023). While we focus on three analytical pipelines in the Results section, it is important to note that this is not an exhaustive list of possible pipelines. There are many examples in the literature of the tools being used in different ways, often combined with novel scripts or external tools to meet unique user needs.

**Table 1:** Overview of software.

Functional category	Name	RRID	Application type	Function	Manual or automated
Atlas-registration	QuickNII	SCR_016854	Desktop	Linear atlas-registration	Semi-automated
	VisuAlign	SCR_017978	Desktop	Non-linear refinement of atlas-registration	Manual
	DeepSlice	SCR_023854	Desktop and Web	Linear atlas-registration	Automated
	WebAlign	SCR_026758	Web	Linear atlas-registration	Semi-automated
	WebWarp	SCR_026759	Web	Non-linear refinement of atlas-registration	Manual
Atlas-based analysis	Nutil	SCR_017183	Desktop	Atlas-based analytical tool	Semi-automated
	QCAlign	SCR_023088	Desktop	Tool for performing quality control steps	Manual
	QuickMask	SCR_016854	Desktop	Create masks for hemibrain analysis	Semi-automated
	QuickMaskNL	SCR_017978	Desktop	Create masks for hemibrain analysis	Semi-automated
Preprocessing	CreateZoom	SCR_026625	Web	Conversion of images to pyramid format	Automated
Viewing sections and results	SeriesZoom	SCR_026908	Web	Viewer	N/A
	LocaliZoom	SCR_023481	Web	Viewer and atlas-based analytical tool	Manual
	MeshView	SCR_017222	Web	Atlas-viewer	N/A

### Software for image registration to a reference brain atlas

#### QuickNII

QuickNII is a standalone desktop application for user-guided affine spatial registration of brain section images to a 3D reference atlas (Puchades et al., 2019). A key feature of the software is its ability to generate user-defined cut planes through the atlas templates that match the orientation of the cutting plane of the 2D experimental images, thereby generating adapted atlas maps. The reference atlas is transformed to match specific anatomical landmarks in the corresponding brain section images. Such landmarks are exemplified in the user manual^2^ and in this resource. In this way, the spatial relationship between the brain section image^3^ and the atlas is defined, without introducing transformations in the original image. Following registration of a limited number of sections containing key landmarks, transformations are propagated across the entire image series. As the propagation relies on the numbering of the section images, it is important that the sections are named using the file naming convention (_sXXX). The propagations should be validated and saved by the user for each section, with application of fine positional adjustments as required.

On the architecture level, QuickNII consists of two co-located executable components implemented in two programming languages for historical reasons. The graphical user interface (GUI) is implemented in MXML+ActionScript (runs on Adobe Integrated Runtime, which is bundled as “captive runtime,” requiring no installation). A slicer service running in the background is implemented in Java (and has a bundled JRE requiring no installation). The two components communicate via standard output and local TCP connections (using the loopback interface).

#### VisuAlign

VisuAlign is a monolithic desktop application for applying user-guided nonlinear refinements (in-plane) to an existing, affine 2D-to-3D registration, such as one created using the QuickNII software (Gurdon et al., 2024). While linear registration tools are vital for bringing experimental image data to standardized coordinate spaces, for precise quantitative analysis residual anatomical variability among test subjects after registration must be addressed. VisuAlign displays the section images with atlas overlays established using QuickNII, allowing users to make manual adjustments by positioning anchor points on the atlas overlays, which can be dragged to their correct anatomical position. VisuAlign uses Delaunay triangulation over the target points (final position of the anchor point), calculates barycentric coordinates for each target pixel inside their containing triangle, and then uses the same coordinates in the source triangle to sample the original (linear) atlas slice.

VisuAlign is implemented in Java, with the graphical user interface built using JavaFX and FXML. While internally there are components and modules, like a slicer from QuickNII; the binary distributables are compiled with J-Link. This both eliminates the need for installing a separate Java Runtime and radically reduces the size of the VisuAlign package, at the same time rendering the internals inaccessible to the outside world. Thus, VisuAlign offers no other interfaces than the actual files that users load and save.

#### DeepSlice

DeepSlice is a deep neural network, trained to predict the position of coronal mouse brain sections within the Allen mouse brain Common Coordinate Framework version 3 (CCFv3) (Wang et al., 2020). DeepSlice is provided as both a Python package (without a GUI) and as a web-application, allowing users to choose between a full featured version of the application and a simplified web user interface which prioritizes ease of use. DeepSlice automatically produces registration files that can be opened directly with QuickNII and VisuAlign, allowing users to inspect the registration result and to make positional adjustments as required. It performs a linear registration and does not predict non-linear deformations. DeepSlice has been validated with assessment of performance relative to expert alignment in the DeepSlice article (Carey et al., 2023).

#### WebAlign

WebAlign is a new web-application for user-guided affine spatial registration of brain section image data to a 3D reference atlas, with similar functionality to the QuickNII desktop application, but enables use of histological section images at original high-resolution for performing registration as opposed to downscaled images supported in QuickNII. A key feature of the tool is its ability to generate user-defined cut planes through the atlas templates (atlas maps) that match the orientation of the cutting plane of the 2D experimental images. Primarily it is a client-side web-application, running in a browser. Implementation languages are HTML5/CSS for the user interface, and JavaScript for processing. Requirements are deliberately kept low. Thus, WebAlign is expected to work properly with web browsers released in March 2017 or later.^3^

The server-side part of WebAlign consists of a very short PHP routine (10 instructions) implementing OIDC token exchange with the EBRAINS IAM service. This step is expected to remain necessary when integrating WebAlign into other environments, although the actual implementation language can be changed. Other requirements for the hosting server are minimal, at the time of writing, WebAlign requests whole files from its hosting server (compressed atlas packages are entirely transferred to the client).

Storage infrastructure of user data for WebAlign is integrated with the EBRAINS “Data-Proxy”^4^ that provides an S3-like interface. Adapting to similar infrastructures is expected to be straightforward. Feature requirements are again low since WebAlign can already operate with down/uploading whole objects at a time. WebAlign can use HTTP RANGE requests if users decide to store their DeepZoom images into ZIP archives (DeepZoom images may consist of several millions of small files, and they can be inconvenient to handle individually).

#### WebWarp

WebWarp is a new web-application for nonlinear refinement of spatial registration of section images from rodent brains to reference 3D atlases, with similar functionality to the VisuAlign desktop application but enables working on high resolution histological section images. WebWarp is compatible with registration performed with WebAlign. Technical details for WebWarp are identical to WebAlign: they operate on the same data and pose the same requirements toward both the browser and the backend (hosting server for the application and storage infrastructure for user data).

### Software for atlas-based analysis

#### Nutil

Nutil is a desktop application for pre-processing of histological brain section images which are typically large and difficult to process with standard image analysis software (Groeneboom et al., 2020). Is it also a key component of the QUINT workflow (Yates et al., 2019), allowing quantification of labeled features in regions defined by a reference brain atlas. It thereby incorporates functionality for transforming, renaming and converting formats of large image files (Transform feature), and for quantifying features in regions defined by an atlas using output of QuickNII or VisuAlign combined with images segmentations generated with a tool such as ImageJ, ilastik or QuPath (Quantifier feature). It is a standalone Windows 64-bit application written in Qt C++, with many new features and atlases implemented since its initial publication in 2020 (detailed in the release notes on Github).

#### QCAlign

QCAlign is a desktop application for performing quality control steps for serial section images and atlas-registration, enhancing the quantitative analysis conducted with the QUINT workflow (Gurdon et al., 2024). It allows the screening of section images for damage, and the assessment of the quality of the atlas-registration as performed with QuickNII and VisuAlign by manual systematic sampling methods. It also enables users to explore the reference atlas hierarchy and to define a customized hierarchy level to use for the assessments.

It is implemented in JAVA and has a similar user interface to VisuAlign, implemented in JavaFX and FXML. Internally it shares large amounts of code with VisuAlign: the two applications share their data descriptor format, the images and atlas overlays, which they load and display in a similar way (QCAlign works with both linear and nonlinear registrations). However, the actual functionality has a completely distinct implementation. New modules are implemented for enhanced atlas management (QCAlign allows users to customize atlas-granularity via a collapsible hierarchy-tree), for generating, pre-filling and interaction with a sampling grid, and for creating the statistical output.

#### QuickMask

QuickMask is a new standalone software with a basic GUI, enabling users to automatically generate hemisphere masks corresponding to brains sections registered to a reference atlas using QuickNII or VisuAlign. QuickMaskNL generates hemisphere masks respecting nonlinear deformations applied around the midline with VisuAlign. The customized masks are directly compatible with the Nutil software and can be used to perform separate quantifications of labeling in the right and left hemisphere in brain sections using the QUINT workflow. Preliminary versions of QuickMask and QuickMaskNL are available for download through the NITRC page for QuickNII and VisuAlign, respectively.

### Viewers and related tools

#### CreateZoom

CreateZoom is a new web-application for converting individual 2D images (.tiff; .jpeg or .png) to image pyramid files in Deep Zoom format (DZI). It is a prerequisite for workflows using WebAlign and WebWarp and for creating SeriesZoom and LocaliZoom viewer links. The application consists of a back-end service for concurrent batch creation of Deep Zoom Images via PyVips, a wrap of the lower-level image processing library libvips (Cupitt et al., 2025). The front-end allows users to define location and to select files to process. Internally, the application handles all actions asynchronously, to schedule processes and poll status of tasks, allowing the freedom to scale variables for the host. The storage service for such pyramid image files is connected from the EBRAINS “Data-Proxy” application accessed with a token acquired from the identity and access management service (IAM) at EBRAINS (see text footnote 4). Processed images are compressed into .dzip files ready for further use.

#### SeriesZoom

SeriesZoom is a new viewer, the most basic online 2D image viewer we provide, allowing viewing of all DZI images (e.g., created by CreateZoom), from a single location. It does not show any atlas overlay or require any kind of series descriptor; it simply collects all DZI images it finds at a provided location. A filmstrip is provided with small icons, and the selected image is displayed in a pan-and-zoom fashion. SeriesZoom is also used as a brain section image viewer on the EBRAINS data sharing infrastructure for publicly shared dataset.

SeriesZoom is implemented using HTML5, JavaScript, and CSS and runs entirely in the browser. It needs access to a series of DZI images. The current implementation assumes S3-like storage, with support for listing objects with a prefix. The discovery function can be easily adapted to different systems providing similar listing functionality. If there are no options for discovery, it can instead be modified to fetch an actual text/JSON with the list of DZI images.

#### LocaliZoom

LocaliZoom is a new web-based pan-and-zoom 2D image viewer coupled with a volumetric atlas slicer, and a navigational aid showing the entire brain section image series as a “filmstrip.” Building on the open standard DZI format, it efficiently visualizes large brain section images in the gigapixel range, allowing image zoom from common, display-sized overview resolutions down to the microscopic resolution without downloading the underlying image dataset. In addition, LocaliZoom has an annotations and extraction feature. Markers can be manually placed by the user through the GUI, these markers are converted to point clouds easily visualized in the 3D viewer, MeshView and can be downloaded as csv files. LocaliZoom is also used as a brain section image viewer on the EBRAINS data sharing infrastructure for publicly shared dataset which have been registered to a reference atlas using QuickNII or VisuAlign.

LocaliZoom is a 100% client-side web application (HTML5, JavaScript, CSS); it is the base of WebWarp, without the need for a read-write storage system. Thus, its structure and requirements are similar to WebWarp. LocaliZoom needs images in DZI format, and a series descriptor that contains their relation to anatomical atlases. While LocaliZoom is simpler than WebWarp, it is also more flexible, a single “configuration.js” allows tuning all aspects of the application related to accessing data (series descriptor, DZI descriptor, DZI tiles), that already enabled us to use LocaliZoom with multiple image storage strategies (internal solution, swift object storage, EBRAINS “Data-Proxy,” individual tiles or tiles bundled into a single file), and descriptor formats (QuickNII, VisuAlign, WebAlign/WebWarp).

#### MeshView

MeshView is a new web-application for real-time 3D display of surface mesh data representing structural parcellations from volumetric atlases, such as the Waxholm Space Atlas of the Sprague Dawley Rat Brain.

MeshView runs entirely in the web browser; and besides HTML5, JavaScript, and CSS, uses WebGL (a variant of OpenGL that is designed web environment, and programmable from JavaScript). MeshView is the most resource-hungry web application. One aspect is the network traffic it generates. While we provide highly optimized and compressed meshes, even the simplest atlas with 80 anatomical regions (WHS SD Rat version 2, from 2015; Kjonigsen et al., 2015) starts its operation by downloading 56 megabytes of data, and other atlases are much larger. The tool also supports loading point clouds (both as URL parameter and entered manually by the user), and the size of point cloud descriptors can also go up to tens or hundreds of megabytes.

The other resource-requiring aspect is rendering. Solid and transparent rendering of anatomical regions is fast (solid rendering uses simple Phong shading, and transparent rendering does not have to deal with complex order-independent algorithms as users find too many structures distracting and simply switch them off). Point cloud rendering is reasonably performant; users can adjust point size on a per-cloud basis. Lowering point size to a single pixel for clouds with huge amounts of points usually works well, but computers with integrated graphics solutions may experience slowdowns. The most complex feature is the support for cutting meshes as if they were solid objects. The algorithm is simple and refined. Thus, the “even-odd rule” for filling closed polygons is extended to 3D (and thus filling polyhedra), and the cut plane (described by a point and a normal vector) is used to leave the surface open, effectively in “odd” state, stored in the stencil buffer. Almost every aspect of the algorithm is fast, but the stencil buffer must be restored to its initial, clear state between rendering each anatomical structure. We find that this single step is taxing for integrated graphics processors.

#### Additional scripts

Some additional scripts are available for QuickNII^5^ related to the propagation algorithm used during image registration to the reference atlas.

#### Python scripts

Python scripts for analyzing point clouds related to the second example of use in the results part are available on Github.^6^

#### Overview of atlas versions currently available

Our applications have been extensively tested and support the following murine brain atlases: the Allen Mouse Brain Atlas CCF version 3, delineations from 2015 and 2017 (Wang et al., 2020); the Kim Unified Mouse Brain Atlas (Chon et al., 2019); DeMBA, a developmental mouse brain atlas for ages P4–P56 (Carey et al., 2025) and the Waxholm Space Atlas of the Sprague Dawley rat v2 (Kjonigsen et al., 2015), v3 (Osen et al., 2019), v4 (Kleven et al., 2023). In addition, some alternative atlases have been compiled in our tools.^7^

## Results

To date, our software suite includes 13 applications, which have been cited in more than 130 original research articles, ranging from studies of tract tracing experiments to classical histological studies, and to studies of cleared brains captured by light sheet microscopy. As the tools are modular, they can be combined into unique analytical pipelines to meet the needs of a range of study paradigms. The QUINT workflow, combining the use of several of our desktop applications, is the most cited of these pipelines and supports whole-brain mapping of labeled features in brain section image series from mice and rats (Yates et al., 2019). We now also offer web-based pipelines, integrated with the EBRAINS research infrastructure, facilitating standardized analysis of data in an online environment. Here, we first outline new developments in the desktop applications and how they can be combined to perform brain-wide analysis using the QUINT workflow. We then describe the web-applications and how they can be used for transparent sharing of histological data and for performing analysis using a reference brain atlas. Finally, we exemplify three analytical pipelines using our software by their use in research studies from the literature. The studies have unique experimental design, requiring different solutions and analytical approaches. Some of these were collaborative projects, which allowed us to build new software and add new functionality in response to requests from our collaborators. We highlight the advantages of using the EBRAINS solutions in each case and point to limitations and areas for future development. While the pipelines exemplified here are commonly used, this is not an exhaustive list of possible pipelines for which our tools may be relevant. There are many examples in the literature of the tools being combined in different ways or with external software or scripts to meet unique user needs [for some examples, see (Lubben et al., 2024; Nemeth et al., 2024; Timonidis et al., 2024; Vatsa et al., 2024)].

### Desktop applications

The QUINT workflow as originally presented in Yates et al. (2019) combined the use of three desktop applications supporting whole brain mapping of labeled features in brain sections from mice or rats. The core software in the workflow is QuickNII (Puchades et al., 2019) for performing registration to a reference brain atlas, ilastik (Berg et al., 2019) for segmenting the images to identify features for quantification, and Nutil for performing whole-brain mapping and regional feature quantification (Groeneboom et al., 2020). The QUINT workflow has since been developed further with new releases of the core software offering new functionality and additional atlases, increased compatibility with alternative open-source software, and has been extended with new software.

We have added the possibility to use VisuAlign for refining the atlas registration achieved using QuickNII, by allowing users to manually adjust regional boundaries as applied to the section image planes. This step is optional, but is commonly needed to achieve accurate registration, especially for sections that are distorted, damaged or that differ anatomically from the reference atlas template. For fully flexible registration methods that support manual adjustment of regional boundaries, the quality of the atlas-registration will dependent on the anatomical expertise of the researcher performing registration and the time they invest to make refinements (see our illustrated guide of anatomical landmarks for registration)^9^. As such, validation must be carried out for histological series on an individual basis. Acknowledging the need for efficient methods of validation, we developed the QCAlign software for performing quality control steps using manual systematic sampling methods. QCAlign is useful for screening the section images for damage and for assessing the quality of the atlas registration achieved using the workflow, which is important for validating research findings. As an additional feature, QCAlign can also be used to define customized compilations of reference atlas regions to use for regional quantification, enabling greater flexibility (Gurdon et al., 2024). Optional functionality is provided by DeepSlice using convolutional neural networks, providing an automated registration of coronal mouse brain sections to the Allen CCFv3 atlas (Carey et al., 2023), compatible with the workflow, which can be refined using QuickNII and VisuAlign as needed. For image segmentation and detection of labeled features, software such as QuPath (Bankhead et al., 2017), Cellpose (Pachitariu and Stringer, 2022) or Fiji (Schindelin et al., 2012) are now compatible with the workflow.

### Online web-applications

Following up on feedback and requests from our users through the EBRAINS user support service, we have developed web-versions of our registration software, WebAlign (for linear registration) and WebWarp (for non-linear registration), available as Collaboratory apps on EBRAINS. These build on the same code base as our desktop applications. However, they are novel in that they are integrated with the EBRAINS research infrastructure for data sharing and are compatible with our new online viewers for histological sections and atlases (SeriesZoom, LocaliZoom and MeshView), thereby providing new analytical possibilities and opportunities for transparent sharing of research findings.

The main advantages of the web-applications are simplification of data management and file preprocessing steps, which are a challenge when analyzing large cohorts of mouse or rat brains. The file management is streamlined, enabling image series uploads via the Data Proxy service and conversion of the image files to pyramid files by the CreateZoom app. Taking advantage of the EBRAINS core services for user identification (IAM) and the Collaboratory service,^8^ users get access to a private working space, where they can upload their datasets to a private bucket and use analytical pipelines, where image registration to a reference atlas of mouse or rat brain is the first step. We provide a public demonstration collab to guide users.^9^ After a brief introduction on how to setup all the web-applications and convert the image files to pyramid files (DZI), the online registration is done interactively in the web browser. All the results are saved in the Collaboratory storage bucket and can be opened in the WebWarp application for refinement with non-linear registration. WebWarp also uses the DZI file format, which allows users to zoom-in to high-resolution, to better decipher region boundaries and achieve a more precise registration. Furthermore, EBRAINS users can open datasets that have been publicly shared through EBRAINS by fetching a link on the dataset card. By opening this link in WebAlign, the image series is automatically created and saved as a WebAlign file (.waln) in the storage bucket. Users can then modify the registration or apply non-linear corrections using WebWarp. All the web-apps are designed to be interoperable and compatible with other analysis tools. They allow users to compare different datasets and combine the results from multimodal experimental setups, thus increasing the reusability of data.

The LocaliZoom app enables visualization of brain section image series with atlas overlays, allowing users to manually annotate and extract point coordinates which can be visualized in the MeshView app together with atlas meshes. In addition, high-resolution microscopy viewers such as SeriesZoom and LocaliZoom provide a seamless user experience and can generate image service links for datasets shared via the EBRAINS data sharing service, upon request by the data provider.

### Example analyses

#### Mapping of efferent connections in the rat brain

In a recent article, Reiten et al. (2023) shared a large data collection from tract tracing experiments in rats aimed at investigating the connections between brain regions involved in spatial navigation, decision making and working memory. The collection consisted of serial coronal sections from 49 rat brains in which the anterograde tracers Biotinylated dextran amine (BDA) or *Phaseolus vulgaris*-leucoagglutinin (Pha-L) were injected in the orbitofrontal, parietal or insular cortex (Kondo and Witter, 2014; Olsen et al., 2017; Mathiasen et al., 2015; Olsen and Witter, 2016; Olsen et al., 2019). Because publicly available rat connectivity datasets are scarce, the authors prepared the data and metadata for sharing through the EBRAINS data portal, with the collections shared as three datasets: projections from the insular cortex^10^ (Mathiasen et al., 2020); projections from the posterior parietal cortex^11^ (Olsen et al., 2020); and projections from the orbitofrontal cortex^12^ (Kondo et al., 2022).

**Figure 1:**
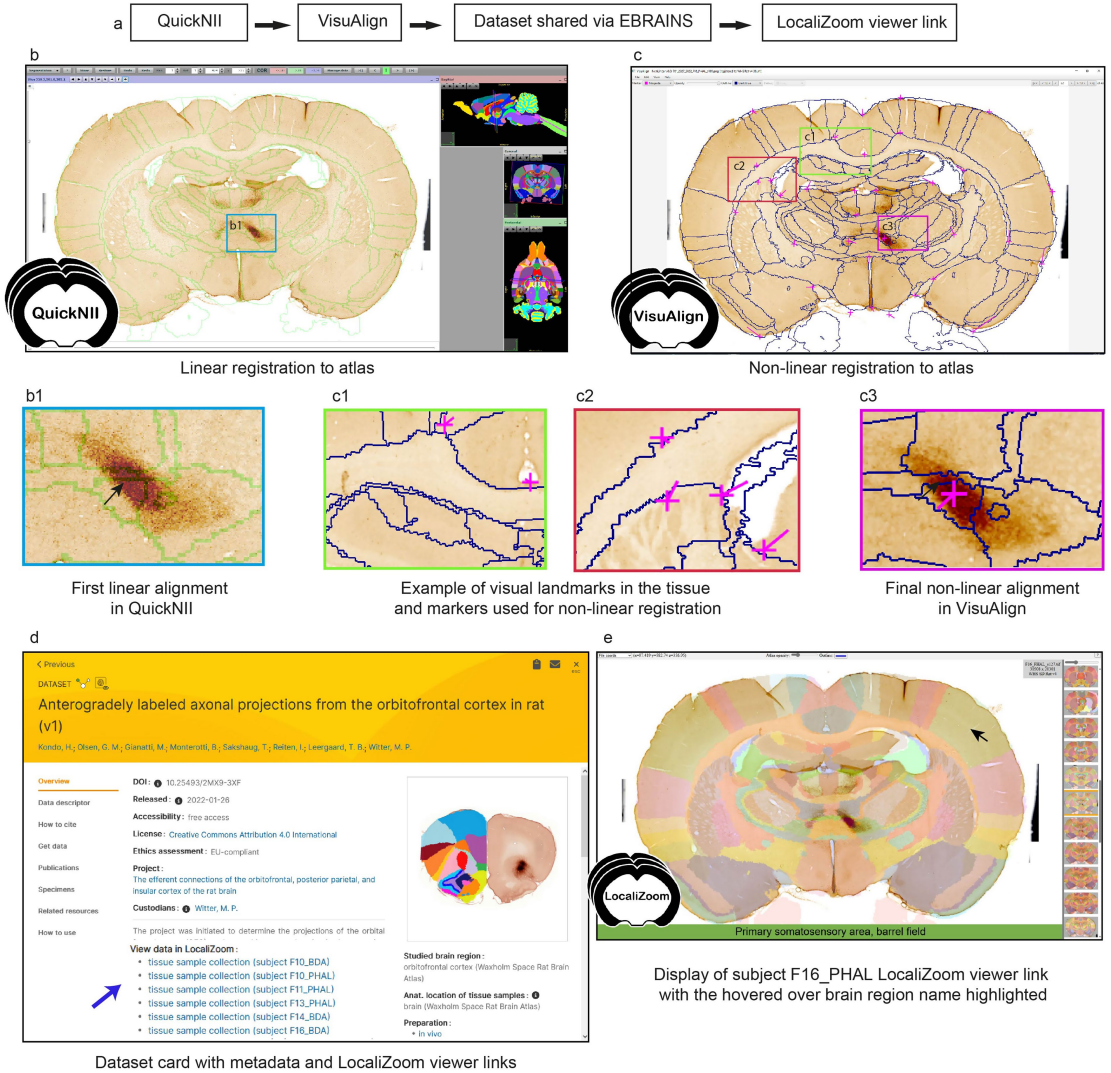
**(a)** Workflow for section image registration to a reference atlas and creation of LocaliZoom viewer links. **(b)** Section image registration with the QuickNII software and **(c)** VisuAlign software. **(c1,c2)** Tissue landmarks are used to guide the non-linear registration. Comparison of registration before **(b1)** and after **(c3)** non-linear adjustments illustrates the importance of this step for correct alignment of small brain regions. **(d)** In this case, the atlas registrations were made available on the EBRAINS dataset card via the service link provided through the LocaliZoom app. **(e)** LocaliZoom service link displaying the whole brain section image series with the atlas overlay visible and brain region name is displayed when hovering the mouse on it for ease of identification. The data used here for illustration is from subject F16_PHAL in dataset https://doi.org/10.25493/2MX9-3XF.

To increase interoperability and opportunities for combined analysis and reuse of the datasets, thereby improving FAIRness (Wilkinson et al., 2016), all section images were registered to the 3D Waxholm Sprague Dawley rat atlas version 4 (Kleven et al., 2023) using the registration software QuickNII (Puchades et al., 2019) and VisuAlign. The workflow is presented in Figure 1a. First, pre-processing steps were applied to ensure that the section images had a proper orientation and sequential positioning along the antero-posterior axis by at least two researchers. Then, linear registration of the images to the 3D atlas was performed in QuickNII for a global positioning (Figure 1b), before refinement of the registration using in-plane non-linear deformations with VisuAlign (Figure 1c). Visual landmarks in the tissue are used to guide these deformations (Figures 1c1,c2). As illustrated in Figure 1, the non-linear adjustment of the atlas to match the experimental sections was essential to achieve correct positioning of the labeled fibers, especially for smaller brain regions or specific nuclei, like the submedius thalamic nucleus (Figures 1b1,c3). The registration software finally allowed users to create atlas overlays on their images, downloadable as 2D images. These registration processes still rely on manual work and good knowledge of brain anatomy by the users. The development of automatic registration algorithms for rat brain series would be a welcome addition to this workflow.

For datasets published on EBRAINS (Figure 1d), data providers have the option to request viewer links available from the EBRAINS data cards for displaying the section images with atlas overlays (Figure 1e). The viewer links are created using our image viewer LocaliZoom, and allow users to (1) browse through the whole image collection arranged in antero-posterior sequence; (2) zoom in on particular regions in the experimental images for closer inspection as they are stored in the pyramid file format DZI; (3) explore the images with or without the atlas overlays visible at different intensities; (4) share the viewer links with their collaborators and colleagues.

#### Mapping projections in the mouse brain

In this example, the authors were interested in the topographical organization of the first link in the neuronal projections from the cerebral cortex to the cerebellum: the corticopontine projections (Ovsthus et al., 2024). To study these connections in mice, they used publicly available serial brain section images, which had connections revealed using injection of fluorescently labeled anterograde tracer molecules. Images from the Allen Mouse Brain Connectivity collection and datasets published on EBRAINS were combined^13^ (Ovsthus et al., 2024; Tocco et al., 2024).^14^

**Figure 2:**
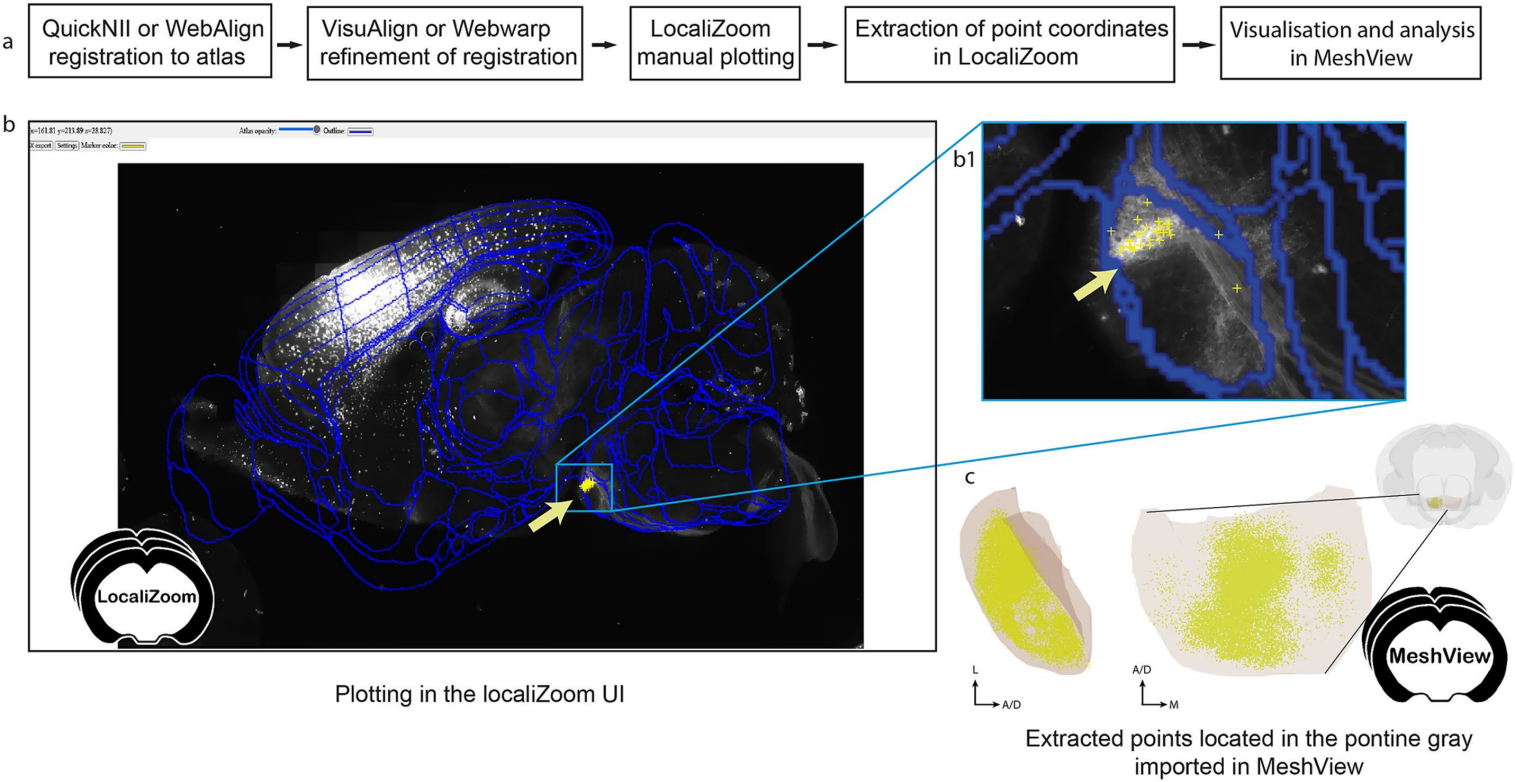
**(a)** Workflow for extracting point clouds from registered brain section series. **(b)** The LocaliZoom app displays the original experimental images after they have been converted to a pyramid format (DZI). **(b1)** Users can zoom in and out of high-resolution images. The atlas-registrations are imported from a JSON file generated by the registration software (either QuickNII/VisuAlign or WebAlign/WebWarp) and can be visualized as contour lines or colored brain regions with different levels of transparency using a transparency slider button. The color of the outlines can be changed by the user. **(c)** Extracted points can be visualized in MeshView. The data used here for illustration is from subject littermate control 11643_13 in dataset https://doi.org/10.25493/11HT-S4B.

The workflow is illustrated in Figure 2a. To extract information about the location of these projections, all the brain section images were registered to the Allen mouse brain Common Coordinate Framework (CCFv3_2017) using the QuickNII software. The location of the axonal terminal fields in the pontine nuclei was recorded manually using a local instance of LocaliZoom (RRID: SCR_023481; https://localizoom.readthedocs.io) for assigning point coordinates at regular intervals (Figure 2b). This feature in LocaliZoom allows users to place markers on the objects-of-interest and thereby capture their coordinates (Figure 2b1). The plotting reflects the observed density of labeling. Users can easily navigate through their image collections using the filmstrip view (Figure 2b). A manual process for extracting coordinates was shown to be more precise in this case than a more automatic process using image segmentations (e.g., the QUINT workflow) as too much non-specific labeling was included [for details see (Ovsthus et al., 2024)].

**Figure 3:**
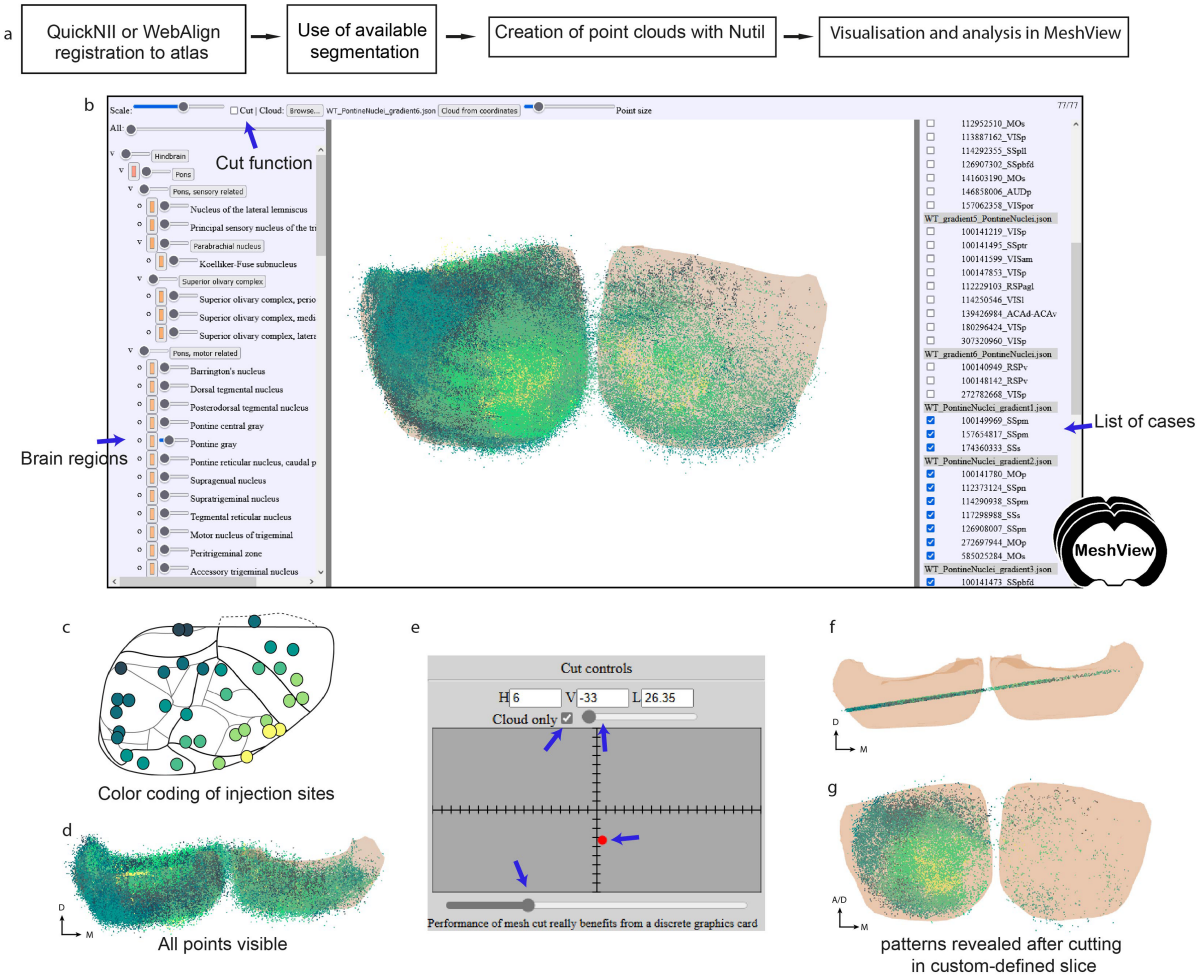
**(a)** Workflow for extraction of point clouds from brain section series and analysis of topographical patterns in MeshView. **(b)** The main UI of MeshView is shown in with selection of the brain region mesh on the left side (arrow pointing to the pontine gray region). The meshes have different degrees of transparency and are color-coded according to the atlas hierarchy label. These colors are editable by the user. The list of uploaded point cloud files (JSON format) is shown on the right side and can be interactively toggled on and off. The cutting feature is found on the top left. **(c)** A schematic of the cortical tracer injection sites is shown where the positions of the injections have been color-coded from yellow to dark green based on the topographical location (see github repo https://github.com/Neural-Systems-at-UIO/3d-point-clouds for more details). **(d)** Same point cloud as in **(b)** viewed from above illustrates the density of points. **(e)** Panel showing the “double cut” feature implemented in MeshView for this specific study. It enables the creation of point cloud slices for an easier inspection of the topographical patterns **(f,g)**. The example data shown here are point clouds showing spatial distribution of corticopontine projections originating from 35 tract-tracing experiments in wild type mice (https://doi.org/10.25493/GDYP-B1B).

The markers representing the labeling were manually applied section by section in LocaliZoom, exported as lists of point coordinates, and visualized as point clouds by uploading the LocaliZoom output file directly to the 3D viewer MeshView (RRID: SCR_017222; details; https://meshview-for-brain-atlases.readthedocs.io/en/latest/; Figure 2c). Some special features like the double cut, and region-specific versions were added to MeshView to aid visualization of patterns in the terminal distributions located in the pontine gray region across several animals (Figure 3).

When large point clouds from many animals are co-visualized (Figures 3b,d) it is difficult to discern spatial distribution patterns. By color-coding point clouds according to the position of injection sites in the cortex, it becomes possible to explore different patterns of topographical organization (Figure 3c). Jupyter notebooks with Python scripts were shared with the datasets allowing users to reproduce these patterns or apply the method to their own data (see the Methods part for more details). Additionally, a double cut feature was added to MeshView to visualize the point clouds as a slice (Figures 3e,f); thereby several different distributions patterns could be demonstrated like fan-like or concentric distributions (Figure 3g; see Ovsthus et al., 2024 for more details).

In addition, for using the double cut feature, we provide MeshView links for specific regions like the caudoputamen only or hindbrain only.^15^

This type of analysis can be done using the web applications WebAlign, WebWarp and LocaliZoom in the EBRAINS Collaboratory (see text footnote 9).

To our knowledge, no similar workflow is available today. Although it involves manual steps, the main advantage is the possibility to extract signal where automatic image segmentation algorithms fail, i.e., when the signal to noise ratio is too low or when objects are too densely packed like the cell somas in the hippocampal principal layers. The workflow is also well suited to compare locations of user-defined objects across series of sections mapped to the same atlas, allowing efficient comparison of object locations across images cut in different angles.

#### Characterizing mouse brain composition in a complex high-throughput study

**Figure 4:**
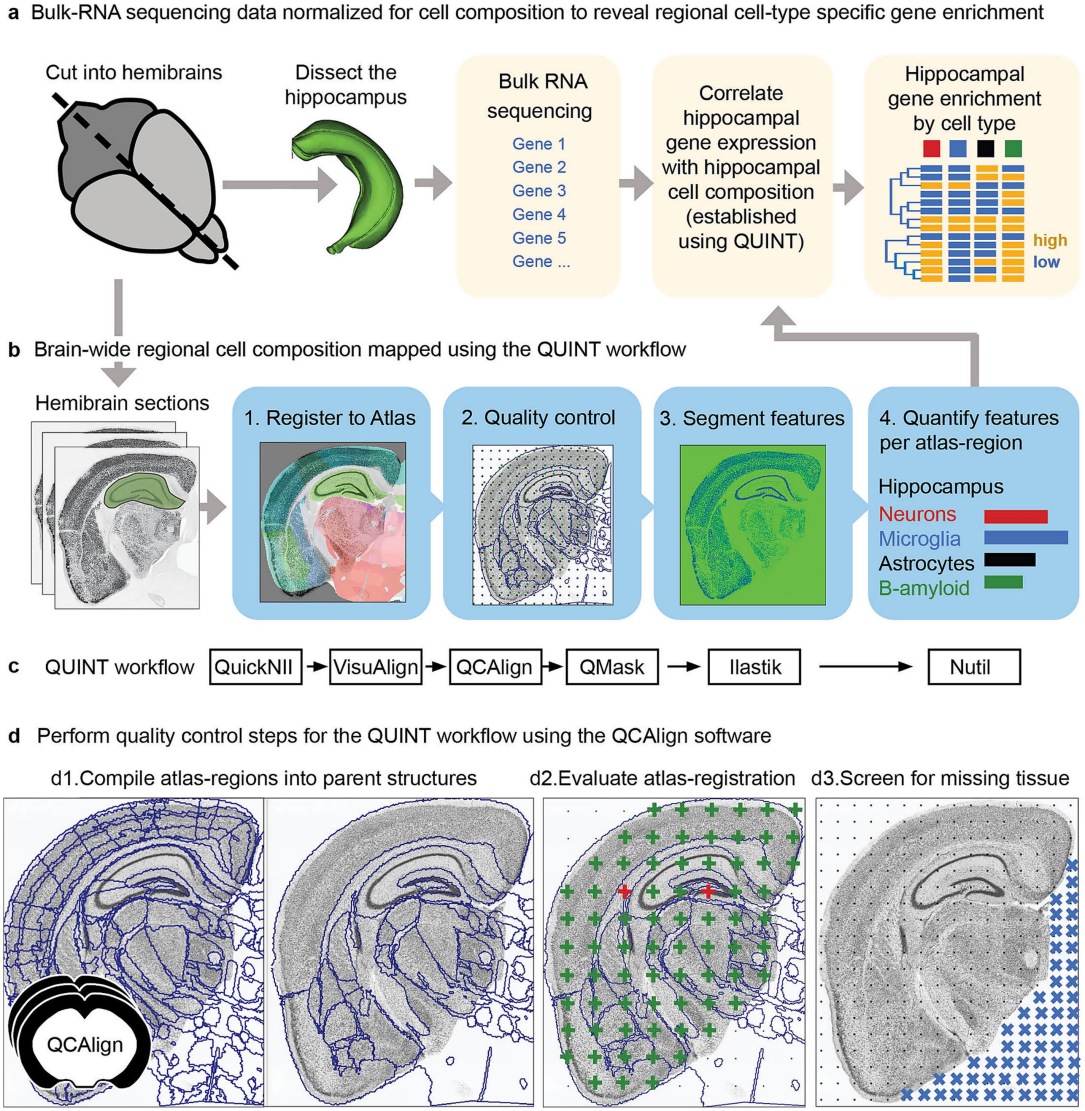
Forty AD-BXD mice were used to explore resilience mechanisms in Alzheimer’s disease using transcriptomic and histological methods. **(a)** To reveal cell-type specific gene set enrichment patterns in the hippocampus, brains from the 40 mice were divided into hemibrains, with one hemibrain microdissected, supplying tissue for bulk-RNA sequencing. The bulk-RNA sequencing data was correlated with cell composition data from the hippocampus from the other hemisphere, established using the QUINT workflow. By integrating the bulk-RNA sequencing data with histological results from the other hemisphere, candidate genes and pathways involved in resilience to disease could be revealed in a region-specific as well as cell-type specific manner **(b,c)**. To explore cell composition using a reference brain atlas, histological data from one hemisphere was analyzed using the QUINT workflow **(c)**, quantifying neurons, microglia, astrocytes and beta-amyloid. This involved four key steps: 1. Registration to a reference brain atlas using QuickNII and VisuAlign. 2. Quality control steps using QCAlign with creation of hemibrain masks using QuickMask. 3. Image segmentation using ilastik to identify the features-of-interest. 4. Quantification of the features-of-interest in reference atlas regions using Nutil. **(d)** To ensure reliable histological results, the quality control software, QCAlign, was developed and integrated in the QUINT workflow providing functionality for **(d1)** establishing parent regions for enabling quality control of the atlas-registration and to use for quantification of features, **(d2)** for evaluating the quality of the atlas-registration using systematic sampling (green crosses indicate correct registration, red crosses indicate incorrect registration), and **(d3)** for establishing regions and sections to exclude from analysis due to damage (missing tissue represented by blue crosses).

In a recent study, Gurdon et al. (2024) investigated the effect of genetic makeup on the development of Alzheimer’s disease using a novel mouse model (AD-BXD), with a goal to reveal resilience mechanisms. The mouse model incorporated genetic diversity with AD risk mutations: resulting in strains with variable symptomatology despite carrying identical high-risk mutations (Neuner et al., 2019). In an exploratory study of multiple AD-BXD strains, brains from 40 mice were divided into hemibrains: with one hemibrain dissected to supply tissue for bulk-RNA sequencing (Figure 4a). The other hemibrain was sectioned, labeled and imaged to allow histological regional brain-wide mapping of neurons, microglia, astrocytes and beta-amyloid (Figure 4b).^16^ Due to the exploratory nature of the histological study, there was a need for a comprehensive analysis method supporting regional comparison across ages and strains. The use of a reference atlas to define regions for analysis supports such an exploratory approach. However, accurate registration is critical, and can be difficult to achieve and verify, especially for high-throughput studies involving genetically diverse animals as the anatomy of experimental models may differ from reference animals. Brain sections may also be distorted, damaged or torn by the sectioning process.

In collaboration, we established the BRAINSPACE project for making the QUINT workflow applicable for such high-throughput applications (Gurdon et al., 2024). The QUINT workflow provided the functionality for bringing features into a common reference space: with registration to the Allen CCFv3 performed with QuickNII, with VisuAlign used to perform the manual adjustments needed to achieve a good anatomical fit over the sections, matching the outer edges as well as internal boundaries made visible by labeling (Figure 4c).

A limitation was a need to validate the region boundaries to be used for quantification due to the expected anatomical differences between individual mice. This validation was challenging, since not all regions included in the reference atlas could be identified in the section images of this study. To bypass this, reference regions of the atlas were combined into parent regions with visible boundaries in the section images. These parent regions were in turn used as boundaries for quantification. We developed the QCAlign software for compiling reference regions into suitable parent regions (Figure 4d1), and for comparing these regions to boundaries visible in the section images by manual systematic sampling using anatomical expertise (Figure 4d2). By utilizing QCAlign, we were able to confirm accurate registration of 77 parent regions across the whole brain, which were then used for quantification of labeled features. We also used QCAlign for rapidly screening sections for damage, allowing systematic removal of sections and regions with more than a set damage percentage (Figure 4d3).

By combining the use of VisuAlign for refining the atlas-registration with QCAlign for establishing regions with visible boundaries to use for quantification, for validating the atlas-registration to these regions, and for performing checks for tissue damage, we were able to counter the distortion and damage that is common in histological studies, ensuring reliable results. As the coronal sections originated from one hemisphere only, the QuickMask software supplied customized hemibrain masks tailored to each section and required by Nutil for hemibrain analysis.

By establishing regional cell composition in this study, results could be compared across ages and strains. Furthermore, hippocampal regional cell compositions could be correlated with hippocampal bulk-RNA sequencing data, allowing candidate genes involved in resilience and progression of AD to be revealed in a region-specific as well as cell-type specific manner (Figure 4b). Thus, by integrating transcriptomic data with regional cell composition data, resilience pathways could be revealed and localized to specific cell-types. While this was a pilot study, focusing on the hippocampus for the development of the integration method, it demonstrates the power of integrating different data modalities using location as a common denominator.

## Discussion

To date, the software summarized here has been employed across a diverse spectrum of published studies, encompassing a range of animal models and experimental methods, and catering to a variety of output requirements. These studies have in common that they are based on 2D brain sections and aim to map the section images into a 3D common reference space. This integration facilitates data comparisons and lays the foundation for future discoveries. It also supports a more nuanced understanding of the brain and its diseases (Bjerke et al., 2018). We have showcased three examples of use of the software to meet challenges unique to different experimental setups. In the first, the desktop applications, QuickNII and VisuAlign are used for performing atlas-registration, enabling creation of LocaliZoom service links for displaying rat tract-tracing image series in the Waxholm Space atlas through the EBRAINS data sharing platform. The second demonstrates mapping of cortico-pontine connections in the mouse, with extraction of point coordinates of the terminal fields using the LocaliZoom application and study of topographical patterns using the MeshView software. Finally, the third demonstrates comprehensive brain-wide mapping of mouse brain data in a high-throughput context using the QUINT workflow, allowing location-based integration of data of two different modalities (histological and transcriptomic data).

Our desktop applications have been widely used by the research community, with the QUINT workflow used to (1) provide brain-wide counts of various cell types, receptors and pathological markers in brain sections from traditional histological studies (Lubben et al., 2024; Geertsma et al., 2024; Bjerke et al., 2025; Guardamagna et al., 2025; Telpoukhovskaia et al., 2022; Bjerke et al., 2021; Bjerke et al., 2022; Jo et al., 2022; Kim et al., 2021; Yao et al., 2022), (2) to investigate brain connectivity in tract-tracing experiments (Timonidis et al., 2024; Yao et al., 2022; Poceviciute et al., 2023; Ham and Augustine, 2022; Tocco et al., 2022; Whilden et al., 2021), and (3) to map markers in tissue clearing experiments captured by light sheet microscopy (da Silva Correia et al., 2024; Lopes et al., 2022). While our software was not developed specifically for light sheet microscopy data, a 2D to 3D registration method has some advantages over 3D-to-3D methods for this data type. This is because clearing procedures induce deformations in the tissue, which can be challenging to adjust for using volumetric registration approaches, ultimately affecting the subsequent registration result. Automated registration methods can be tailored to specific tissue clearing techniques (Perens et al., 2021); however, the ability to interactively refine the atlas-registration using visual landmarks, as implemented in VisuAlign, is a clear advantage as it can be easily adopted without coding ability (da Silva Correia et al., 2024). Furthermore, the QUINT workflow provides a means for transparent analysis as the atlas-registration can be shared with the data and used with our web-applications (SeriesZoom and LocaliZoom) to create shareable microscopy viewer links with atlas overlays, as demonstrated for several datasets shared through the EBRAINS data sharing platform (ebrains.eu).

While most published studies have used our software as described in our user documentation, innovation is at play, with a proportion developing their own scripts and methods used in concert with the EBRAINS software to solve problems unique to their own experimental design. For example, the Henderson group have combined the use of QuickNII, VisuAlign, QuickMask and Nutil with the popular histopathology toolbox QuPath (Bankhead et al., 2017) for identifying features-of-interest, creating a modified version of the QUINT workflow for fluorescence (Lubben et al., 2024; Geertsma et al., 2024; Goralski et al., 2023). They have openly shared this method as an iprotocol.^17^ By sharing these scripts and methods, the research community benefits from access. Obstacles to analysis are also revealed, which can prompt developers to implement new features in their software, continuing the development cycle. Software development is thereby a community effort, with no clear start and end point. However, by developing analytical tools in concert with a coordinated research infrastructure, as we have done here through the EBRAINS research infrastructure, we have been able to develop more mature research software, with more community involvement, than would have been possible without the research infrastructure approach. We have been able to offer web-applications through a research infrastructure for user authentication, resource allocation and data storage. We have also reached a larger target audience and have been able to offer a better user support service, which has provided the feedback needed to drive developments.

Currently, our applications incorporate two adult mouse brain atlases [Allen CCFv3 (Wang et al., 2020) and Kim Unified Mouse Brain Atlas (Chon et al., 2019)], one adult rat brain atlas (WHSSD) (Kleven et al., 2023), and a developmental mouse brain atlas for ages P4–P56 (DeMBA) (Carey et al., 2025). Brain atlas development is a growing field with new atlases being released and existing atlases extended on a continuous basis. To assist developments using these resources, the BrainGlobe initiative provides an overview of available atlases for small animal models and have created an Atlas API which compiles these atlases and their metadata as a resource for developers^18^ (Claudi et al., 2020). We are collaborating on future API developments, with potential to expand our atlas repertoire to match this collection in future releases. Co-development efforts such as these are critical to ensure compatibility of software across research groups and institutions; and to develop and adopt the standards needed to maximize the impact of such developmental efforts.

In a future development, we plan to release the entire QUINT workflow for brain-wide mapping in an online workbench environment. This will provide a streamlined user experience and ameliorate issues relating to file preparation and transfer between software, which can complicate offline workflows. It will also allow users to create shareable microscopy viewer links, increasing the FAIRness (Wilkinson et al., 2016) of the datasets and related analyses. While online solutions have clear advantages over downloadable software, they also have downsides as they require on-going maintenance and are more prone to issues due to multiple dependencies. By providing both online and offline solutions using standardized atlases that are interoperable with solutions for transparent data sharing, our software promote data FAIRness and facilitate scientific discovery and data reuse.

## Data Availability

Publicly available datasets were analyzed in this study. This data can be found at: https://search.kg.ebrains.eu/?facet_type[0]=Dataset&category=Dataset.

## Footnotes

^1^ https://ebrains.eu

^2^ https://quicknii.readthedocs.io/en/latest/index.html

^3^ https://zenodo.org/records/7575515

^3^ https://developer.mozilla.org/en-US/docs/Web/API/Fetch_API

^4^ https://wiki.ebrains.eu/bin/view/Collabs/the-collaboratory

^5^ https://github.com/Neural-Systems-at-UIO/QuickNII-extras

^6^ https://github.com/Neural-Systems-at-UIO/3d-point-clouds

^7^For details see https://quint-workflow.readthedocs.io/en/latest/QUINTintro.html#supported-atlases.

^9^ https://zenodo.org/records/7575515

^8^ https://wiki.ebrains.eu/bin/view/Main/

^9^ https://wiki.ebrains.eu/bin/view/Collabs/image-registration-and-analysis-demo

^10^ https://doi.org/10.25493/WK4W-ZCQ

^11^ https://doi.org/10.25493/FKM4-ZCC

^12^ https://doi.org/10.25493/2MX9-3XF

^13^ https://doi.org/10.25493/GDYP-B1B

^14^ https://doi.org/10.25493/11HT-S4B

^15^See https://meshview-for-brain-atlases.readthedocs.io/en/latest/displaying.html for more details.

^16^ https://doi.org/10.25493/SZ0M-EE6

^17^ https://dx.doi.org/10.17504/protocols.io.4r3l22y6jl1y/v2

^18^ https://brainglobe.info/
